# A Novel Bioinspired PVDF Micro/Nano Hair Receptor for a Robot Sensing System

**DOI:** 10.3390/s100100994

**Published:** 2010-01-26

**Authors:** Fei Li, Weiting Liu, Cesare Stefanini, Xin Fu, Paolo Dario

**Affiliations:** 1 The State Key Lab of Fluid Power Transmission and Control, Zhejiang University, Zhejiang Province, Hangzhou, 310027, China; E-Mails: li_fei@zju.edu.cn (F.L.); xfu@zju.edu.cn (X.F.); 2 CRIM Lab, Polo Sant’Anna Valdera,Viale Rinaldo Piaggio 34, 56025, Pontedera (Pisa), Italy; E-Mails: cesare@sssup.it (C.S.); dario@sssup.it (P.D.)

**Keywords:** artificial hair receptor, micro/nano fiber fabrication, aligned PVDF micro/nano fiber, thermo-direct drawing

## Abstract

This paper describes the concept and design of a novel artificial hair receptor for the sensing system of micro intelligent robots such as a cricket-like jumping mini robot. The concept is inspired from the natural hair receptor of animals, also called cilium or filiform hair by different research groups, which is usually used as a vibration receptor or a flow detector by insects, mammals and fishes. The suspended fiber model is firstly built and the influence of scaling down is analyzed theoretically. The design of this artificial hair receptor is based on aligned suspended PVDF (polyvinylidene fluoride) fibers, manufactures with a novel method called thermo-direct drawing technique, and aligned suspended submicron diameter fibers are thus successfully fabricated on a flexible Kapton. In the post process step, some key problems such as separated electrodes deposition along with the fiber drawing direction and poling of micro/nano fibers to impart them with good piezoeffective activity have been presented. The preliminary validation experiments show that the artificial hair receptor has a reliable response with good sensibility to external pressure variation and, medium flow as well as its prospects in the application on sensing system of mini/micro bio-robots.

## 1. Introduction

**Figure 1.**
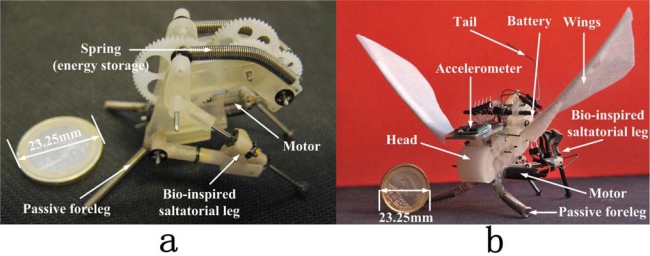
Jumping robot prototypes; (a) GRILLO II has a bioinspired leg design, it shows a good jumping performance but an unstable flight after takeoff [10], (b) prototype GRILLO III is optimized by mass center readjustment and the optimization of the passive forelegs in order to improve the stability during jumping, flight, and landing.

Focusing on mini/micro autonomous robots, lessons from Nature often provide us inspiration for useful solutions and suggest ingenious designs for small bio-robots. In the past, many different bioinspired robot prototypes have been built in the search for efficient and stable solutions for robot gaits. Among them, legged locomotion is preferred by most designers, rather than the simpler wheeled designs, because of its high efficiency, low energy consumption and better stability on un-structured or tough terrains [1] achievable with biped [2–4], quadruped [5,6], to hexapod [7,8] and even octopod configurations [9]. Based on this consideration, we designed our jumping robot prototypes GRILLO II & III (Figure 1a,b) inspired by the small jumping insects known as leafhoppers. Thanks to scale effects and the characteristics of jumping locomotion, this jumping-robot, as a millimeter-sized mobile robot prototype with integrated power supply offers a lot of advantages because of its lesser energy consumption and better practicability [10].

After finishing the mechanical design, the work is now focused on the feasibility of the sensing and control systems in order to improve the jumping stability (e.g., wing control during gliding) and to make the robot react to the environment (e.g., flow turbulence detection). Still learning from the natural world, we start from the investigation of the hair cell sensory receptors, which perform as primary mechano-transducers in both the auditory system and the vestibular system of vertebrates. In mammals, the hair cell receptors are located in the cochlea and play an important role in the perception of sound, while in fishes and amphibians, they are located within the lateral line organ to detect the surrounding water motion. Hair cells possess a characteristic organelle which consists of tens of hair-like stereo-cilia. So-called hair bundles are able to pivot around their base when a force is applied to the tips [11–16].

**Figure 2.**
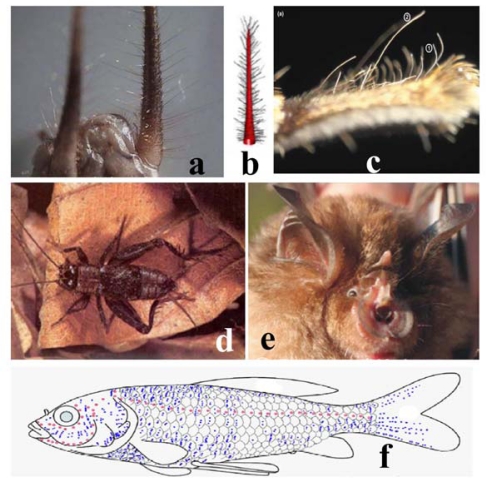
Hair cell receptor existing in many animals; (a, b, d) cricket cerci [17], (c) a spider tarsus [18], (e) bat hearing system [19], (f) goldfish (*Carassius auratus*) lateral line system [20].

Sensory hairs widely exist in the natural world (Figure 2). For arthropods, high performance detection systems composed of mechano-receptive cuticular hairs are evolved to sense the slightest air displacement around them, such as that generated by approaching predators. A cricket’s abdomen is covered with mechano-receptive cerci which are sensitive to those slight air currents generated by a wasp’s wings or a toad’s tongue. Such sensory hairs alert the insects when a predator is sneaking around them, and give them a chance to escape from predation [13,17]. The adult tropical wandering spider (*Cupiennius salei*) has hundreds of trichobothria on its ambulatorial legs and pedipalps ranging from 20 to 1,500 μm in diameter. It was also found those sensory hairs in different length are able to mechanically couple with different frequencies and receive the medium vibration generated by flying insects.

Various artificial hair cell (AHC) prototypes based on piezo-silicon cantilever structures have been developed in the past. The feasibility of fabricating an AHC on a silicon substrate through normal lithographic fabrication and the PDMA (plastic deformation magnetic assembly) method was demonstrated [14,21]. Polymer AHC cilia were obtained by patterning SU8 cylinders on silicon piezo-resistive sensors [22]. An all-polymer AHC was fabricated by depositing carbon-impregnated polyurethane force sensitive resistors (FSRs) at the base of vertical cantilever polyurethane cilia. The FSRs transduced the motion of cilium into resistance changes [23]. Another type of interesting high aspect ratio SU8 sensory hairs on a silicon nitride membrane based on capacitive change measurements was presented in [15,24]. The SU8 hairs, up to 1 mm long, underwent a deflection due to flow momentum, which led to basilar disc rotation and change in the capacitance between the nitride membrane and the substrate.

The artificial hair receptors mentioned above all suffer from several drawbacks. First, the fragility of substrate materials limits their application in microsystems and makes them unpractical in the engineering field. As an example, micro bio-robot design frequently involves curved surfaces on which those fragile rigid substrate materials are not easily applied. Second, all these artificial hair prototypes are used to transduce force/momentum to their respective sensitive elements (piezo-resistive or capacitive) instead of being sensitive themselves. That means some other accessories besides the sensory hair itself are necessary, thus leading to an additional fabrication burden and a complicated structure which causes additional difficulties in miniaturization and increases the costs. Furthermore, the aspect ratio of the polymer artificial hairs made by mold techniques and lithographic patterning (usually no more than 20) is not enough compared with its animal counterparts.

In this paper the authors address a novel artificial hair receptor (PVDF micro/nano fiber) being sensitive itself, and placed on a flexible substrate with a high aspect ratio and small size, similar to its animal counterpart. This artificial hair is fabricated by a direct fiber drawing technique, by using a micro-syringe pump to push a PVDF solution through a glass micropipette and draw on a substrate.

## 2. PVDF Hair Receptor Modeling

### 2.1. A biological Model of Hair Receptor

**Figure 3.**
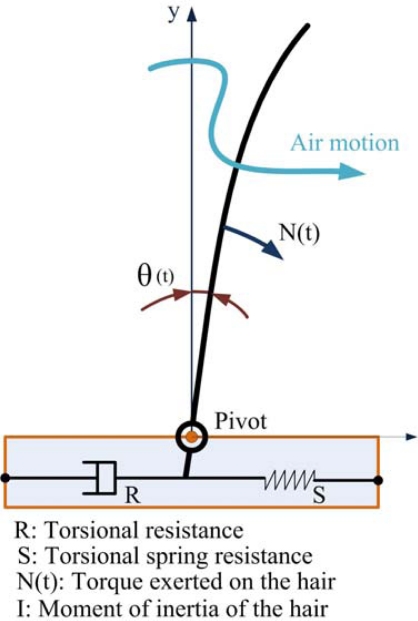
Inverted pendulum model of hair cell (Shimozawa *et al*. [12]).

The mechanism, morphology, and modeling of the hair cell type mechano-receptor system such as cricket cercal wind receptors have been unveiled thanks to biological research [12]. The hair is modeled as an inverted pendulum (Figure 3).

It can be described by a second-order mechanical system which is determined by the spring stiffness *S*, the momentum of inertia *I* and the torsion resistance *R*. For the angular momentum:

(1) $$I\frac{d^{2}\theta \left(t\right)}{{\mathrm{dt}}^{2}}+R\;\frac{d\theta \left(t\right)}{\mathrm{dt}}+S\;\theta \left(t\right)=N\left(t\right)$$

The hair is deflected by the drag force on the air shaft due to the airflow surrounding the cercus. The total external torque *N(t)* can be calculated by the integration of the drag force along the hair shaft:

(2) $$N\left(t\right)=\int_{0}^{L_{0}}\;F\left(y,\;t\right)\cdot y\cdot \mathrm{dy}$$

### 2.2. A Simplified Model for PVDF Artificial Hair Receptor

Even though PVDF has shown good mechanical and physical performance in some past research, it is still difficult to entirely duplicate the structure of a biological hair receptor (a vertical free-fix structure) due to the limitations in drawing fabrication and post process techniques. Considering the convenience in our drawing method and the feasibility in electrodes fabrication, a fix-fix structure is selected at the first step. By improving the techniques of fabrication and fiber post-processes, an entirely biological hair structure will be viable in our future work.

The PVDF fiber generates the charges on the electrodes while deformed by the flow based on the piezoelectric activity. The generated charge density is:

(3) $$D=\frac{Q}{A_{e}}=\frac{Q}{\pi \mathrm{rL}}=d_{3h}\;X$$

where *D* is the charge density; *Q* is the charge; *A_e_* is the effective electrode area; *r* is the radius of hair fiber; *L* is the length of the artificial hair (aligned micro/nano PVDF fiber); *d_3h_* is the piezoelectric coefficient constant which is the combination of *d_31_*, *d_32_* and *d_33_* in our case of in fiber drawing direction, transversal and frontal section direction respectively; *X* is the stress.

**Figure 4.**
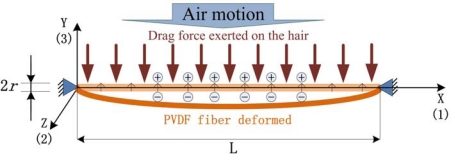
A simplified model of an artificial PVDF hair receptor.

Figure 4 describes the case that the artificial hair receptor is placed in the unidirectional air flow and used as a flow sensor. While the flowing direction is perpendicular to the hair sensor shaft, the drag force exerted on the hair can be calculated as follows:

(4) $$F_{d}=C_{D}\;\left(\frac{1}{2}\;{\rho v}^{\alpha }\;A\right)=C_{D}\;\left({\rho v}^{\alpha }\;\mathrm{rL}\right)$$

where *F_d_* is the drag force; *ρ* is the fluid density; *v* is the flow velocity; *A* is the projected frontal area of the hair sensor facing the flow; *r* is the radius of hair fiber; *L* is the length of the artificial hair (aligned micro/nano PVDF fiber); *C_D_* is the drag coefficient that is a dimensionless constant and the value is 1.0–1.3 for a cable or wire in air; the coefficient *α* depends on the Reynolds number. Usually, the drag coefficient is proportional to the square velocity (*α* = 2); but for small values of the Reynolds number (laminar flow), the drag coefficient is inversely proportional to the velocity (*α* = 1).

The piezoeffective activity is the combination of contributions *d_31_*, *d_32_* and *d_33_*, but *d_31_* is the main contributing parameter due to the PVDF high compliance property. Thus the deformation in the y-direction generated by the pulling stress along the x-direction is ignored in order to simplify the model. Therefore, considering only the primary deformation along x-direction and ignoring the effect in z-direction (*i.e.*, gravity force), charges on the electrodes generated by the air motion can be obtained as:

(5) $$Q=Q_{31}=\pi \;{\mathrm{rLd}}_{31}\;X_{1}$$

where *Q_31_* is the charge generated by the tensile stress in x-direction and *X_1_* is the average tensile stress in this direction due to the fiber deformation.

The average tensile stress can be calculated approximately from the length change of the fiber in the x-direction. Combined with the drag force and piezoeffective equations, in the case that the drag works completely as the pulling force due to the compliance of the PVDF fiber, we have:

(6) $$Q_{31}=\pi {\mathrm{rLd}}_{31}\;X_{1}=\left(\pi {\mathrm{rLd}}_{31}\right)\cdot \frac{L^{\prime }-L}{\mathrm{LE}}=\left(\pi {\mathrm{rLd}}_{31}\right)\cdot \frac{\Delta L/L}{E}$$

where *L′* is the fiber length after deformation, *ΔL* is the length change. Assuming that all deformation discussed is in the linear elastic range, then the classical equation of deflection curve can be used here. Thus the length change *ΔL* can be described as follows:

(7) $$\Delta L=\int_{\Gamma }\;\mathrm{ds}-L=\int_{0}^{L}\;\sqrt{1+{f^{\prime }}^{2}\;\left(x\right)}\mathrm{dx}-L$$

(8) $$f\left(x\right)=\frac{F_{d}\;x^{2}}{24\mathrm{LEI}}\left(x^{2}-2\mathrm{Lx}+L^{2}\right)$$

where *Γ* is the deflection curve of the deformed fiber; *f(x)* is the equation of the deflection curve; *E* is the Young’s modulus of PVDF material and *I* is inertia moment of the cross-section.

Assuming that the two electrodes on the single PVDF micro/nano fiber are along the axial direction with a small separating gap, the average distance between the electrodes *s̄* is:

(9) $$\bar{s}=\frac{4r}{\pi }$$

Thus the fiber capacitance can be simplified as:

(10) $$C=\varepsilon \;\frac{A_{e}}{\bar{s}}=\varepsilon \;\frac{\pi {\mathrm{rL}}^{\prime }}{\frac{4r}{\pi }}=\varepsilon \;\frac{\pi^{2}\;L^{\prime }}{4}$$

Then we can finally obtain the relationship between air motion and the voltage output *U* of this artificial PVDF hair receptor:

(11) $$\{\begin{array}{c} U=\frac{Q}{C}=\frac{\pi {\mathrm{rLd}}_{31}\cdot \left(\int_{0}^{L}\;\sqrt{1+{f^{\prime }}^{2}\left(x\right)}\mathrm{dx}-L\right)}{\varepsilon \frac{\pi^{2}\;L^{\prime }}{4}.\mathrm{LE}}\\ f\left(x\right)=\frac{C_{D}\;{\rho v}^{\alpha }\;{\mathrm{rx}}^{2}}{24\;\mathrm{EI}}\left(x^{2}-2\mathrm{Lx}+L^{2}\right) \end{array}$$

### 2.3. Scaling Down Analysis

The voltage output on the two electrodes of this artificial hair receptor is growing as the flow velocity increases (e.g., from 0 to 0.1 m/s). According to the previous discussion, the charges on the electrodes generated by fiber deformation are calculated from the longitudinal strain *ΔL/L* which is related to the pressure on the surface generated by the drag force and the inertia moment of cross section. The pressure exerted on the fiber can be described as:

(12) $$P_{s}=\frac{F_{d}}{\pi \mathrm{rL}}=\frac{C_{D}\;{\rho v}^{\alpha }}{\pi }$$

**Figure 5.**
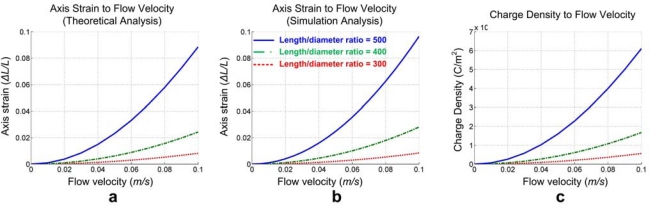
Scaling down analysis; (a) relationship between axial strain and flow velocity with different length/diameter ratio (theoretical analysis), (b) relationship between axial strain and flow velocity with different length/diameter ratio (simulation analysis), (c) relationship between charge density and flow velocity with different length/diameter ratio.

Within a same measurement range (0 to 0.1 m/s for example), *P_s_* increases proportionally to the flow velocity due to the relative fixing of *C_D_* at a small scale. Meanwhile, the axis strain to flow velocity pattern changes dramatically with scaling down of the fibers according to our theoretical analysis and simulation results (Figure 5a,b). The average error between the theoretical analysis and the simulation is about 5%, and mainly results from the model simplification in the CFD and FEA analysis. This result means that the output (charge density on the electrodes) is improved by scaling down the fiber diameter even though it probably leads to an unstable structure of the sensor (Figure 5c).

On the other hand, at low Reynolds number, *α* in equation (4) approximates 1, which means that the drag force is proportional to flow velocity. Then, combined with its high length/diameter ratio profiting from our drawing fabrication technique, the design shows an output voltage-flow velocity pattern with both good strength and linearity.

This leads us to the conclusion that the performance of the artificial hair receptor can be improved by increasing the length/diameter ratio and provides us with the possibility to make full use of the advantages of the novel micro/nano fiber drawing technique to obtain extremely high aspect ratios (up to 10,000) micro/nano fibers.

**Figure 6.**
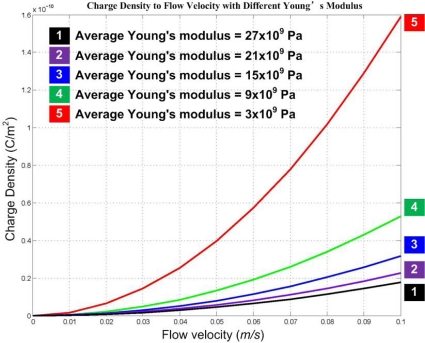
Relationship between charge density and flow velocity with different Young’s moduli.

According to past research, the Young’s modulus of the PVDF material is about 3 × 10^9^ Pa. From the model analysis we find that at the same air velocity, as shown in Figure 6, the output of the hair receptor decreases by increasing the average Young’s modulus of the whole structure (e.g., ranging from 3 × 10^9^ to 27 × 10^9^ Pa) which is tremendously influenced by the thickness of the electrode metal films on the fiber. However, it brings forward the advantages of a larger measurement range because of its better structural strength. So an important issue of the artificial hair receptor design is to find a balance between the signal intensity and the structure strength.

## 3. Aligned Micro/Nano Fibers Drawing Fabrication

### 3.1. Micro/Nano Fiber Fabrication Techniques

Polymeric micro/nano fibers can be processed by a number of techniques such as drawing [25–28], template synthesis [29], phase separation [30], self-assembly [31], electrospinning [32,33] and dry spinning [34]. Continuous thin fibers can be generated by the latter two methods, which have already been extensively used in industrial and laboratory-scale fabrication.

In electrospinning, a suspended dilute drop of polymer is charged with a characteristic voltage (usually hundreds of thousands of volts) that makes the droplet form a Taylor cone. Then a fine jet of polymer releases from the surface of the cone in response to the tensile forces generated by interaction of an applied electric field and the electrical charge carried in the liquid. As a result a bundle of polymer fibers is produced. By directing the jet to a grounded surface, it can be collected as a continuous web of fibers ranging from a few nano to micrometers in diameter.

In dry spinning, the polymer solution, prepared by dissolving the polymer in a volatile solvent, is pumped through a spinneret (die) with up to thousands of holes. After exiting from the spinneret, air is used to evaporate the solvent so that the liquid fibers solidify and then can be collected on a take-up wheel. Fiber stretching provides for the orientation of the polymer chains along the fiber axis, in a meanwhile the solution has to be viscous rather than dilute because of the requirements of the droplet stability.

Continuous fibers can be obtained with both methods introduced above. However, for the former one, fibers are usually generated and distributed randomly on the collector; for the latter one, the produced fibers on the spindles can be collected with some extent of alignment. Unfortunately, producing aligned fibers on designated positions is not feasible in either case. On the other hand, direct drawing and solidifying of micro/nano fibers from volatile solvents can in principle meet this requirement.

As an instrument to form polymer nanofibers, the direct drawing technique was first proposed in 1998 by Ondarcuhu and Joachim [25], who produced fibers of 5 nanometers in diameter and several micrometers in length from specific citrate solutions with an AFM tip. Then, in 2004, Harfenist [26] succeeded in forming suspended fibers between different solid supports by directly drawing between polymer liquid pre-deposited points through nano-instruments. After that, Nain *et al*. [27,28] introduced the glass micropipette into direct fiber drawing. They connected a micropipette to a micro-syringe pump so that they successfully extended the fiber drawing time window by slowly pumping out liquid and succeeded in the fabrication of polymethyl methacrylate (PMMA) and polystyrene (PS) micro/nano fibers.

### 3.2. PVDF Micro/Nano Fiber Drawing

**Figure 7.**
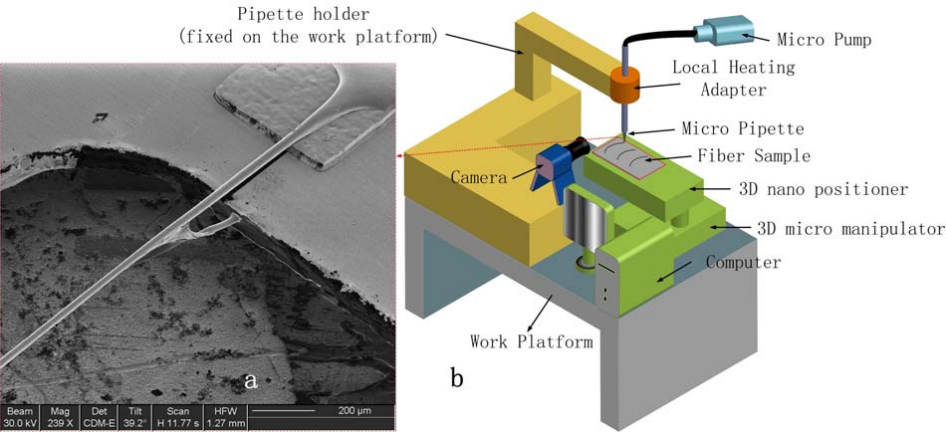
The thermo-direct drawing system; (a) suspended PVDF aligned micro/nano fibers (about 25 μm in diameter) on Kapton (thickness 50 μm), (b) scheme of PVDF direct drawing setup.

The system presented is composed of an automatic 3D nano positioner for defining the drawing path, a 3D micro manipulator used to preliminarily position the micropipette with respect to the video camera, a Sony CXD-V50 video system for monitoring, and a specific designed work platform fixed with the positioner as well as a local heated micropipette fixed on the pipette holder and vertical to the work platform (Figure 7b).

In order to successfully carry on the fiber drawing from a polymer solution, a proper viscoelastic behavior of the material is required because the strong deformation generates stresses during pulling; meanwhile, the cohesiveness should be always balanced with the stresses. Furthermore, drawing processes always accompany with the solidification and transfer the spinning polymer to a solid fiber, which make the situation even more complicated. These difficulties limit the application of direct drawing method in polymer micro/nano fabrication. Only examples of polymeric micro/nano fibers mentioned above have been realized by direct drawing technique.

For PVDF micro/nano fiber manufacture, it has been proven that the dimethylformamide (DMF) is a good solvent for producing PVDF micro/nano fibers in a randomly distributed web with the electrospinning technique [32,33].

**Figure 8.**
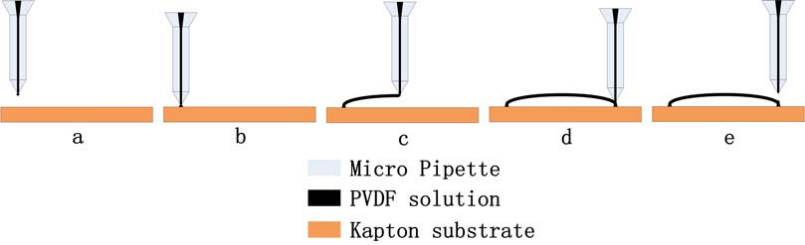
Schematic diagram of the drawing fabrication process of suspended aligned PVDF microfibers.

In our direct drawing technique, the proper PVDF/DMF viscous solution system can be prepared by dissolving 20% weight of polymer in DMF (viscosity 1176 cP) because at this viscosity the droplets on the micropipette tip can remain stable. However, it is still impossible to produce the PVDF micro/nano fibers by a simple drawing method under ambient conditions because of the low evaporation rate and low diffusion ability of DMF, which lead to the formation of a solute skin layer that blocks further solvent evaporation of the PVDF/DMF solution. To solve this problem, a local heating system was applied to the micropipette after design simulation and practice experiments because the elevated temperature can improve both solvent evaporation rate and the polymer mobility. By introducing proper control parameters (temperature, drawing velocity, pump flux, *etc.*), the authors successfully produced a suspended aligned PVDF microfiber array with diameter about 25 μm over a 2 mm gap of a 50 μm thickness Kapton substrate (Figure 7a) [35]. The detailed schematic diagram of fabrication process is shown in Figure 8.

By better adjusting the rheology characteristic property of the solution, the droplet contact area to the substrate and those controllable automation parameters, fabrication of PVDF fibers on a nano scale is also feasible with this novel thermo-direct drawing technique. Right now, in our experience the minimum diameter of the PVDF fibers obtained is about 327 nm.

### 3.3. Post Process of Fiber Fabrication (Electrodes Deposition and Poling)

**Figure 9.**
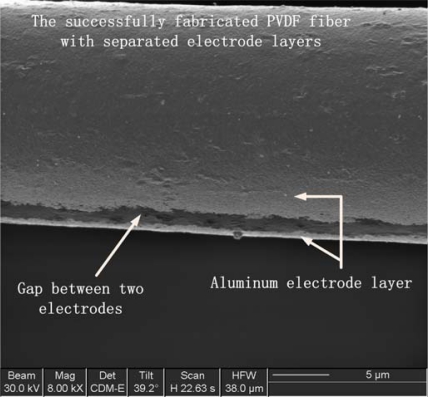
FIB image of the separated electrodes (the gap is about 1 μm) fabricated by thermo evaporation technique on a single PVDF micro/nano fiber.

Electrode deposition is one of the key issues in our fiber post processes. In order to deposit electrode films on both sides of a single micro/nano fiber along with the drawing direction without short circuits, thermoevaporation [36] is used for the electrode fabrication because of its highly collimated deposition path capacity. The fabricated aligned PVDF fibers are fixed on a rigid frame in order to facilitate evaporation on both sides. The successfully fabricated single PVDF fiber with separated electrodes is shown in Figure 9 (the separation gap in the figure is about 1 μm).

**Figure 10.**
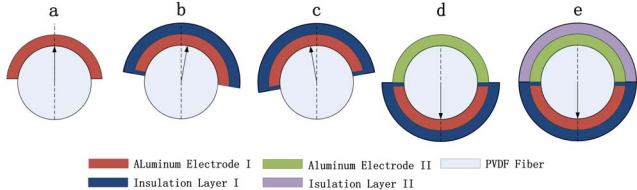
Detailed processing sequence of PVDF micro/nano fibers fabrication; (a) evaporation of the first aluminum, (b–c) by swinging the substrate along the fiber axis, the insulation layer covers the metal electrode completely, (d) fiber inverted in order to evaporate the other aluminum electrode layer. (e) evaporation of the other protection layer.

In this case, metal layers are directly evaporated on both sides of the fiber without any insulation layers which are not necessary because the fiber diameter is still large enough (about 25 μm). However, as the fiber dimension is reduced, a more complicated process including insulated and protection layer fabrication will be needed in the future. An aluminum electrode layer will be firstly evaporated and followed by an insulated layer on this side. By swinging the substrate along the fiber axis, the metal electrode can be covered by this insulation layer completely. Then the frame will be inverted in order to make the other electrode as well as its protection layer. Considering the mechanical requirements, PVDF can be a proper insulator material [37] because of its good dielectric properties and less influence on the mechanical properties of the whole structure after deposition. This detailed processing sequence is shown in Figure 10a–e.

Poling is a very important step to provide the PVDF fiber with a good piezo effect [38]. It is well known that the PVDF material needs to be mechanically stretched before poling in order to transform the non-polar alpha-phase into the polar beta-phase. In the case of micro/nano applications, the stretching procedure is intrinsic in the direct drawing fabrication. Under high electric poling field, the dipoles are aligned. As long as this poling electric field is higher than the coercive field, these oriented dipoles will not go back to a completely random configuration after poling. Actually, the piezo coefficient constants are proportional to the remnant polarization. For our aligned micro/nano fiber array, it is even more difficult to achieve a good poling effect than the case of piezo polymer films due to its tiny size and tremendous spatial nonuniformity.

**Figure 11.**
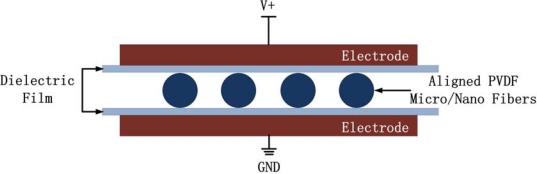
Scheme diagram of an innovated poling device for PVDF micro/nano fiber array.

Figure 11 shows our innovated poling device. The PVDF micro/nano fiber array is placed between two electrode plates and protected from breakdown by dielectric films. The poling voltage applied to the electrodes with a triangular wave is up to 12 KV.

## 4. Validation Experiments

### 4.1. Electronic Interface for PVDF Micro/Nano Fibers

**Figure 12.**
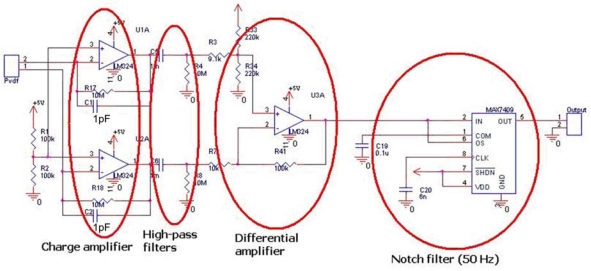
The electronic interface of the PVDF micro/nano fiber sensor.

Since the equivalent circuit of the PVDF piezoelectric material is a charge generator in parallel with a series capacitance, it is necessary to use a high impedance input stage to interface the proposed sensor. Furthermore, parasitic capacitances introduced by connection wires from the sensor to the interface must be eliminated; thus, as the first stage of the electronic interface design, the charge amplifier configuration described in [39,40] and illustrated in Figure 12 was selected. This configuration output is only sensitive to the feedback capacitance rather than the input one. The selected feedback capacitance is 1 pF due to the low value of the PVDF micro/nano fiber capacitance in our case, and the notch filter is 50 Hz in order to eliminate the affection of the line power frequency interference.

### 4.2. Validation Experiments for the Novel Artificial PVDF Fiber Sensor

The validation experiments are firstly designed to verify the capability of the artificial receptor (PVDF micro/nano fiber sensor) to detect the environmental variations such as external pressure changes and the flow turbulence, which are essential for the mini jumping robot sensing systems. And in the future, further precise characterization will be followed.

**Figure 13.**
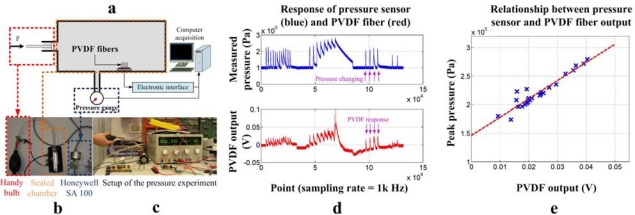
Setup and results of pressure experiment; (a) schematic diagram of the pressure experiment, (b) the handy bulb, the sealed chamber and the Honeywell SA 100 pressure sensor used in the pressure experiment, (c) setup of the pressure experiment, (d) the response of Honeywell pressure sensor (blue) and PVDF fiber sensor (red), (e) relationship between the peak pressure measured by the Honeywell pressure sensor and the PVDF fiber output.

The first validation experiment is built to test the response of the artificial hair receptor to the changes of the external pressure. The experimental setup is shown in Figures 13a–c. The PVDF fiber sample is put into a sealed chamber connected by a reference pressure sensor (Honeywell SA 100) through a small tube to measure the pressure exerted on the PVDF fibers. The pressure variation generated by the handy bulb causes the deformation of the PVDF fibers which results in a voltage output between the electrodes on the fiber (Figure 13d).

As mentioned in the last section, the PVDF equivalent circuit is a voltage source in series with a capacitance or a charge generator in parallel with a capacitance. Therefore the PVDF artificial hair receptor is a dynamic sensor only responding to the pressure variation. Figure 13e shows the relationship between the measured peak pressure in the chamber and generated voltage of a single PVDF fiber which reaches a sensibility of about 0.33 mV/kPa. The result indicates a good sensibility of the artificial hair receptor to external pressure variations, as well as a good linearity of its output pattern. Furthermore, this output magnitude could be multiplied by using a receptor array including tens of nano fibers, which is easily constructed with our thermo-direct drawing technique.

**Figure 14.**
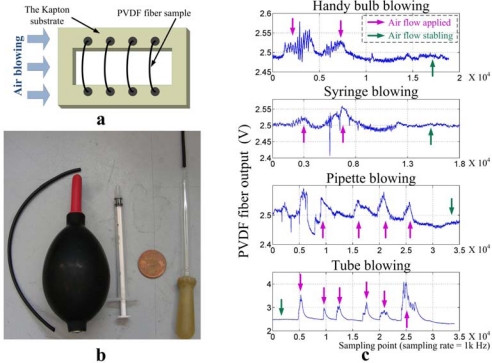
Setup and results of blowing experiment; (a) Scheme of blowing experiments, (b) local blowing tools (the 2 mm inner diameter plastic tube, handy bulb, 1 ml syringe, a comparison coin and a normal micropipette from left to right respectively), (c) fiber response to the blowing by the handy bulb, the 1 mL syringe, the micropipette and the tube from top to bottom respectively.

Then the correlation between the fiber output and different flow turbulences are tested intuitively. The setup diagram of the fiber sample and the local blowing tools are shown in Figure 14a. Various blowing tools are applied during the experiments to generate flow turbulence with different intension and frequencies (Figure 14b). The substrate is fixed on the table in order to avoid its deformation during blowing which may also cause the stretching of the fiber. The results of local blowing experiments shown in Figure 14c indicate that the artificial PVDF hair receptor sample is very sensitive and shows totally different responses to various turbulences. Colored arrows on Figure 13 and Figure 14 indicate the example points on where the pressure changing or the air flow was applied or not.

Results of these two validation experiments have proven the feasibility of PVDF fibers as an artificial receptor for a bio-robot sensing system to detect the wind properties and environmental vibrations, and the contribution of the thermo-direct fiber drawing technique to the sensibility improvement of the micro cilia structure. For example, with ordinary MEMS techniques, the aspect ratio of the cilia structure in [41] is about 7:1, but with the thermo-direct drawing technique, a high aspect ratio of 80:1 is achieved for our PVDF fiber sample. This novel technique brings the hair receptor a very sensitive response to external turbulence and makes it very promising in designing a bio-robot sensing system, not only because of its capability in producing high aspect ratio structure, but also its good “processability” in micro fabrication.

## 5. Conclusions and Future Work

Novel artificial hair receptor prototypes for a jumping mini robot sensing system based on aligned PVDF micro/nano fiber arrays with high aspect ratio have been developed by a novel thermo-direct drawing technique. The key techniques related to the fabrication of the artificial PVDF hair receptor have been presented, including poling and electrode fabrication on the single fiber along the drawing direction. These novel techniques help the piezo materials overcome the limitation of membrane or mat applications and enable a fiber shaped sensor or actuator which is more sensitive and tiny.

The feasibility of the thermo-direct fiber drawing technique on PVDF material and the application prospect of the produced fibers as a sensing system for micro/mini bio-robots has been justified. The validation experiments show a reliable response and good sensibility of the micro/nano PVDF fiber to pressure variations as well as various flow turbulences. Particularly, they indicate that the artificial hair cell receptor is very promising in wind property and environmental vibration detection, which are essential for our jumping mini robot sensing system. Furthermore, it provides a new solution for piezo material based sensors/actuators, besides the membrane study.

Future work will focus on the detailed investigation of the aligned micro/nano PVDF hair receptor. The calibration experiments of the PVDF fiber sample will be carried on in order to find the exact relationship between the ambient environment properties and the fiber output. Then the complete fiber sensor characterization will be tested on a model jumping platform as the next step and finally on a real cricket-like jumping robot. On the other hand, further study on the thermo-direct fiber drawing technique will be also needed. Fabrication of PVDF fibers on a nano scale will be feasible in the future by better adjusting the rheology characteristic property of the solution, the droplet contact area to the substrate and those controllable automation parameters.

In addition, the proposed prototype is only an original design for the convenience in the drawing fabrication. By improving the techniques of fabrication and fiber post processes, an entirely biological structure (a fiber with a fix-free structure) may also be viable in the future. The new type of micro/nano aligned fibers could have more extensive applications, not only on the robot sensing systems, but also in many sensors, actuators and other fields such as tissue engineering, active sensing, neural interfaces, vestibular systems, *etc.*
